# Clinical Practice Models for the Use of E-Mental Health Resources in Primary Health Care by Health Professionals and Peer Workers: A Conceptual Framework

**DOI:** 10.2196/mental.4200

**Published:** 2015-03-23

**Authors:** Julia Reynolds, Kathleen M Griffiths, John A Cunningham, Kylie Bennett, Anthony Bennett

**Affiliations:** ^1^ National Institute for Mental Health Research Research School of Population Health Australian National University Canberra Australia; ^2^ Centre for Addiction and Mental Health Toronto, ON Canada

**Keywords:** translational medical research, professional practice, primary health care, treatment of mental disorders, Internet, health care technology, health promotion, case management, psychotherapy

## Abstract

**Background:**

Research into e-mental health technologies has developed rapidly in the last 15 years. Applications such as Internet-delivered cognitive behavioral therapy interventions have accumulated considerable evidence of efficacy and some evidence of effectiveness. These programs have achieved similar outcomes to face-to-face therapy, while requiring much less clinician time. There is now burgeoning interest in integrating e-mental health resources with the broader mental health delivery system, particularly in primary care. The Australian government has supported the development and deployment of e-mental health resources, including websites that provide information, peer-to-peer support, automated self-help, and guided interventions. An ambitious national project has been commissioned to promote key resources to clinicians, to provide training in their use, and to evaluate the impact of promotion and training upon clinical practice. Previous initiatives have trained clinicians to use a single e-mental health program or a suite of related programs. In contrast, the current initiative will support community-based service providers to access a diverse array of resources developed and provided by many different groups.

**Objective:**

The objective of this paper was to develop a conceptual framework to support the use of e-mental health resources in routine primary health care. In particular, models of clinical practice are required to guide the use of the resources by diverse service providers and to inform professional training, promotional, and evaluation activities.

**Methods:**

Information about service providers’ use of e-mental health resources was synthesized from a nonsystematic overview of published literature and the authors’ experience of training primary care service providers.

**Results:**

Five emerging clinical practice models are proposed: (1) promotion; (2) case management; (3) coaching; (4) symptom-focused treatment; and (5) comprehensive therapy. We also consider the service provider skills required for each model and the ways that e-mental health resources might be used by general practice doctors and nurses, pharmacists, psychologists, social workers, occupational therapists, counselors, and peer workers

**Conclusions:**

The models proposed in the current paper provide a conceptual framework for policy-makers, researchers and clinicians interested in integrating e-mental health resources into primary care. Research is needed to establish the safety and effectiveness of the models in routine care and the best ways to support their implementation.

## Introduction

### Background

Over the past 15 years, there has been a rapidly burgeoning interest in the use of electronic communications to deliver psychological therapies. This has been driven by recognition of the high prevalence of mental health problems and the relatively low treatment rates. For example, up to half of those living in English-speaking developed countries will experience a mental disorder in their lifetime [1-3]. Accordingly, most will directly experience a mental disorder or be in a close relationship with a person who does. Unfortunately, only a minority of people with mental health problems receives treatment. In Australia, treatment rates improved following the introduction of subsidized psychological therapies but still remain substantially lower than those achieved for comparable physical conditions [4].

 E-mental health resources offer a potential means for addressing this treatment gap. Of the different resources, online symptom-focused programs have been the most thoroughly researched. These programs have typically drawn on cognitive behavior therapy (CBT) and involve online modules with interactive components such as symptom questionnaires, multimedia content that can be downloaded, and exercises in which service users practice skills. Programs that incorporate some human support and guidance are referred to as iCBT. Others are designed for use as self-guided, fully automated interventions.

Numerous research trials have shown that Internet-delivered CBT programs can be efficacious and cost effective. A few such programs have accumulated enough evidence to be listed by national review bodies such as the National Institute for Health and Care Excellence (NICE) in the UK and the National Registry of Evidence-based Programs and Practices (NREPP) in the USA. For example, e-mental health programs listed by NREPP include Beating the Blues [5], MoodGYM [6], and the Co-ordinated Anxiety Learning and Management Tools (CALM) program [7].

Further, a recent systematic review found that for three disorders (depression, social phobia, and panic disorder), the evidence for iCBT was sufficient to meet the American Psychological Association’s criteria for an “established” therapy [8]. iCBT interventions have achieved similar outcomes to individually administered face-to-face CBT while requiring much less therapist time [8,9].

While the initial research effort has focused on programs that draw on standard forms of CBT, programs based on other types of therapy can also be efficacious. These include Internet-delivered programs drawing on interpersonal therapy [10], psychodynamic therapy [11], and structured writing techniques [12].

Other technology-based interventions are also accumulating evidence of positive outcomes. Online psycho-education has been shown to improve attitudes to help seeking [13,14] and may be associated with symptom reduction [6]. Information websites can help address the large unmet needs for information, although quality continues to be variable [15,16]. Relapse prevention interventions are also showing promise across service users with diverse diagnostic [17] and/or treatment histories [18].

Other applications include peer-to-peer support [19], game-based interventions [20], mobile apps [21], online chat [22], and video-conferenced therapy [23].

### Implementation in Routine Care

Interest is now turning to the question of how e-mental health resources can be incorporated into traditional systems of care. Primary health care systems are of particular interest because they are typically the first point of contact and provide the bulk of mental health care to individuals, at least in developed countries. In Australia for example, the primary health care system is comprehensive and includes general medical practices, allied health, indigenous health workers, pharmacists, and other services [24]. Incorporating e-mental health services into primary health care could improve treatment rates for common mental disorders, especially for people who are unable or unwilling to access traditional services. It may also generate efficiencies and allow clinicians time to provide more intense care to those who require it [25].

To this end some studies have explored implementation of iCBT in primary health care settings. More research is needed in this area, but positive outcomes have been found for programs designed to treat depression and a range of anxiety disorders [26-28].

In an innovative initiative, the Australian Government has funded the e-Mental Health in Practice (eMHPrac) service to promote key e-mental health resources among primary health care service providers and to train them to use these resources in routine practice [25,29]. These providers include: general medical practitioners; primary care and mental health nurses; indigenous health workers; psychologists; social workers; occupational therapists; counselors; community health workers; and peer support workers.

There are two routes by which e-mental health resources can be incorporated into primary health care (cf [30]).

The best-researched route involves the delivery of iCBT to service users referred to centralized online clinics from community-based service providers. These clinics typically provide some in-house therapist support to service users as they work through programs. Online clinics of this type may receive public health funding to provide free treatment, such as those in Holland and Australia [31,32].

The current paper will focus on the second route, in which community-based service providers incorporate a broad range of e-mental health resources into their practice. This requires clearly differentiated models of practice that are sufficiently flexible and diverse to be relevant to practitioners operating in different contexts and with varying levels of mental health training. Such models must take into account the perspectives and real-life challenges faced by service providers and users in community-based care settings.

Although previous work has considered ways that community-based therapists can use e-mental health resources such as iCBT [33-36], there has been little systematic consideration of models by which diverse providers can incorporate a broad range of e-mental health resources into their practice.

Accordingly, the current paper proposes practice models by which e-mental health resources can be comprehensively incorporated into primary health care systems. The proposed models draw on a nonsystematic overview of published literature and the authors’ experience of training primary care service providers to use e-mental health resources. Our aim in presenting these models is to stimulate discussion, to provide a conceptual framework for future research and to assist clinicians who wish to explore the use of e-mental health resources in their practice.

## Models of Delivery in Primary Health Care

### Broad Approaches

We identify five broad approaches to the use of e-mental health resources, which require varying levels of service provider knowledge and engagement. The five models are: (1) promotion; (2) case management; (3) coaching; (4) symptom-focused treatment; and (5) comprehensive therapy.

Table 1 describes these models, detailing the role of the provider, the role of the e-mental health resource and the contact between the service user and provider. Table 2 shows the workers for whom the models are most relevant and the knowledge required to implement each model.

**Table 1:** Roles of service providers and e-MH resources in each practice model.

Model	Worker provides	Role of e-MH resource	Usual type of contact with worker
Promotion	Information about resources	User decides – can be information, support or treatment	Informal if passive promotion, formal if promotion occurs during a consultation
Case-management	Pre/post assessment, referral to e-resource, crisis support and alternative referral(s) where required	Resource is primary intervention, worker’s role is mainly referral	Pre and post assessments scheduled, service user may initiate additional contact
Coaching	Support to help the person use the e-program Assessment and crisis support may be provided directly by worker or by associated services	Resource is primary intervention, worker’s role is to assist user to engage with and complete program	Assessments, program support likely to be scheduled, service user may initiate additional contact
Integrated into symptom-focused therapy	Individual assessment and formulation	e-resource enhances /extends the work of the therapist in a discrete, symptom-focused intervention	Ongoing and scheduled, service user may initiate additional contact
	Plan and deliver focused therapy incorporating e-MH and human-delivered therapeutic activities.	e-MH programs may also function as a guide for therapists	
Integrated into comprehensive treatment	Comprehensive multi-dimensional clinical assessment and individualized formulation.	e-resource is used flexibly as one part of comprehensive, mixed methods intervention.	Ongoing and scheduled, service user may initiate additional contact.
	Plan and deliver comprehensive intervention incorporating e-MH and traditional therapies.		
	Therapist activities that relate to the e-MH resource may resemble those described in Models 1-4.		

**Table 2:** Service providers most likely to use each practice model and required knowledge.

Model	Likely to suit	Existing practice	Knowledge about e-MH
Promotion	Any worker in a clinical role; workers in non-clinical roles who are supervised by clinicians	Select and provide information about mental health services	Relevant e-MH portals and key information sites
Case management	Any worker in a clinical role that involves screening and referral for mental health concerns	Able to provide screening assessment, alternative referrals and crisis support	Familiar with key e-MH resources and ethical issues relating to e-therapies; able to refer and follow-up appropriately
Coaching	Workers in clinical roles able to assess mental health, refer and support service users’ self-help activity.	As above plus capacity to maintain appropriate and focused coaching relationship.	Familiar with relevant e-MH programs and coaching protocols where they exist, capacity to develop or adapt coaching protocols; familiar with ethical issues relating to e-therapies.
Integrated into symptom-focused therapy	Therapists already providing discrete symptom/ disorder focused therapies such as CBT	Can provide individualized assessment/formulation, deliver symptom-focused therapy in traditional formats (e.g., face to face) and provide access to alternative referrals and crisis support	Familiar with relevant resources and ethical issues
			Able to flexibly integrate e-MH resources into intervention
Integrated into comprehensive therapy	Therapists already providing comprehensive individualized psychological assessment and therapies	Advanced therapy training, capacity to formulate and treat complex problems and use multi-modal approaches	Familiar with available online resources; specific knowledge of e-MH resources and ethical issues relevant to their area of practice

### Referral Models

#### Overview

In the first three models, the e-mental health resource is the main intervention. The role of the community-based provider is to refer the service user to the resource and to provide varying degrees of support.

Advantages for service providers using these models include access to free high quality psycho-educational resources and increased referral options. Use of these models may potentially reduce practice costs and increase effectiveness and efficiency.

#### 1) Promotion

##### What is the Promotion Model?

In this model, information is provided to service users to guide them towards high quality resources. At a minimum, providers can promote credible portal websites, which direct service users to relevant, evidence-based resources [37]. Providers can also become familiar with and promote the resources most relevant to their service - including information sites, symptom-focused treatment programs and online sources of emotional support.

##### Which Providers Are Most Likely to Use the Promotion Model?

Most primary health care providers can promote e-mental health resources. Those in non-clinical roles such as practice managers and administrators can arrange for promotional material to be displayed to service users in waiting rooms or on websites run by the practice. Providers in clinical roles may engage in more active forms of promotion such as briefly describing relevant resources to service users. This may occur in the course of general health consultations as well as in response to service users’ requests for mental health assistance.

For example, general practice nurses have regular contact with people experiencing chronic physical conditions, to whom they provide information, monitoring and treatment. Given the high comorbidity of these conditions with mental health problems, practice nurses are well placed to promote awareness in this at risk group, to detect changes in life circumstances that may increase risk of mental health problems and to respond to the development of symptoms in these service users [38]. Similarly, service providers such as physiotherapists, exercise physiologists, and dietitians treat conditions that are highly comorbid with mental health concerns and can provide information about e-mental health services as appropriate.

There is also enormous potential for promotion by those who provide ancillary services to people experiencing mental health concerns. Community pharmacists are highly accessible and have regular contact with people who are using psychotropic medications. Relationships with pharmacists may be particularly important for people who lack continuity of care in their relationships with other professionals. This may be critical where service users obtain repeat medication prescriptions from different doctors in walk-in clinics.

There have been calls to extend pharmacists’ role in recovery-oriented mental health care [39] and the promotion of e-mental health resources through pharmacies could be one means of facilitating this development. Pharmacists can encourage service users to explore appropriate e-mental health resources in addition to using medication. People interested in self-help interventions may also seek information about supplements such as St John’s Wort. In addition to advice provided directly by the pharmacist, these customers can be encouraged to visit sites that provide evidence-based information about supplements and other interventions.

##### What Knowledge is Required to Use the Promotional Model?

Service providers working in clinical roles will have pre-existing knowledge about mental health, face-to-face care options, and referral processes. These providers would at least need to be familiar with key mental health portals and may also choose to learn about specific resources most relevant to their practice.

Where workers in non-clinical roles engage in promotion activities, it is expected that the clinicians employing them would oversee the selection of resources and the manner in which they were promoted.

##### Current Status of the Promotion Model

This approach requires little specialist knowledge or change to existing practice. In Australia, there is anecdotal evidence that e-mental health resources are already being promoted by mental health service providers and by general health workers in clinical and non-clinical roles including general practice nurses, practice managers, community health outreach workers, physiotherapists, pharmacists, and osteopaths.

#### 2) Case Management

##### What is the Case Management Model?

In this model, the practitioner provides an initial assessment, referral to an e-mental health resource and a follow-up assessment. Referrals are most commonly made to iCBT or self-guided symptom-focused programs, but can also be made to e-mental health support groups and other resources.

Prior to referral, an initial assessment is required to determine the most suitable intervention. E-mental health programs typically use automated screening tools to help guide the service user in their intervention choices. These vary in complexity, but are often focused on the symptoms treated by the particular program. In community-based practice where the service user has approached a service provider for assistance, more comprehensive assessments are required (eg, [40]). These require a relatively high level of skill and service providers engaging in assessment should be appropriately qualified. Assessment will generally occur during face-to-face or possibly video-conferenced consultations [41], although information from e-mental health tools may be incorporated into the assessment.

Once the service user has engaged with the e-mental health program, the provider is not expected to assist them to work through it. However, the provider is expected to provide additional assessment, support, and alternative referrals if the service user does not find the e-mental health program to be helpful, and/or if their condition or situation deteriorates.

When used as an intervention in the case management model, the e-mental health resource is the main psychological treatment for at least one area of concern. It may be provided as a stand-alone intervention used in addition to other interventions. For example, a general practitioner might prescribe psychotropic medication and refer the service user to an iCBT program. Similarly, a person experiencing pain and depression might be referred to a physiotherapist as well as to an e-mental health depression treatment program. E-mental health resources can also be combined with human delivered psychological treatments (see Models 4 and 5 in this paper).

##### Which Providers Are Most Likely to Use the Case Management Model?

This approach is particularly suitable for practitioners who provide non-specialist mental health assessment and referral such as general medical practitioners. It is also used by mental health professionals such as those working as injury / rehabilitation consultants and in community outreach services.

In the case management approach, e-mental health services are considered as referral destinations where appropriate (eg, [40]). This can be particularly useful where specialist assistance is not available in a timely manner or not available at all. For example, we are aware of a public sector mental health team that encourages general medical practitioners to consider this approach for people who have undergone specialist assessment but whose symptoms are not sufficiently acute or severe to meet specialist service admission criteria.

This approach is useful where service users have been accepted into a service but cannot immediately access face-to-face support or psychological therapy, for example where there are waiting lists (eg, [42]). This is highly relevant to services that have existing triage, internal referral, and case management processes such those catering for a particular demographic group (such as young people) or disorder (such as substance use). Workers who already provide assessment and case management in these systems such as youth workers, substance abuse counselors and community health workers, can incorporate e-mental health resources into their work with some additional training.

The case management approach can also be used to introduce e-mental health resources as post-treatment booster or relapse prevention interventions. Anecdotal reports from service users indicate that although they may delay returning to face-to-face therapy until symptoms are relatively severe, they may access e-mental health resources when symptoms are still mild.

##### What Knowledge is Required to Use the Case Management Model?

Service providers using this model will already have clinical roles and relevant skills in assessment and referral. To refer to e-mental health resources, they would also need to be able to locate and evaluate relevant resources and have detailed knowledge of specific resources most relevant to their practice area.

Engaging in active follow-up assessments is critical in this model to enhance safety by ensuring that services are matched to clinical need and by providing access to alternative care as required. It also helps providers to understand how service users in their particular setting respond to e-mental health resources. If outcomes and adherence are disappointing, this should be investigated so that local service users’ preferences can be understood and referral practices can be modified. However, it should be noted that people who do not complete online programs are not necessarily dissatisfied with the program (eg, [43-46]).

##### Current Status of the Case Management Model

This model is currently promoted and supported by a range of online services. For example, Australian online clinics support referrals and case management by offering clinicians’ resources such as an online referral facility [32] and online decision-making tools and guides [45,47,48].

Some online providers have developed referral aids in the style of prescription or referral pads and distributed them to clinicians so they can direct service users to their particular programs. These aids have been distributed in printed format and in an electronic format that can be incorporated into medical practice software (eg, [47,48]).

As part of the eMHPrac project in Australia [29], referral aids have been developed to raise practitioner awareness of multiple resources. These aids are currently incorporated into a software application embedded within clinical information software, and it is hoped in the future that they will be incorporated directly into the most frequently used clinical information software packages (Personal communication by J Proudfoot and J Tennant, 2014).

#### 3) Coaching

##### What is the Coaching Model?

In the coaching model, human support is added to a symptom-focused e-mental health program. The treatment team thus has three components—the service user, the human service provider and the e-mental health program. The online program is the primary intervention and provides the content and structure for the treatment. The coach supports the self-therapeutic activities undertaken by the service user and helps them engage with and complete the program [49].

Coaching combines the benefits of human support and Internet-delivered content. Online formats can reliably deliver material with high fidelity and may enhance learning and retention of critical material compared to traditionally delivered CBT [50,51]. On the other hand, human coaches may improve the match between service user and intervention through assessment and tailoring; support users to complete the program and enhance users’ understanding and application of program material [41,52]. Coaching may be used to reduce the amount of clinician time required to deliver an intervention, improve fidelity and support non-expert workers to deliver basic interventions.

##### Which Providers Are Most Likely to Use the Coaching Model?

The provision of coaching by community-based providers raises critical questions about qualifications, training, and supervision. Requirements will depend upon the service users involved, the programs used, and the degree of therapeutic skill required by particular coaching protocols.

In community-based primary health care settings, coaching is most likely to be delivered by providers with an existing clinical role. For example, in Australia, coaching is likely to be delivered by health professionals but in other countries such as the United Kingdom, coaching is likely to be delivered by para-professional workers such as the IAPT’s Psychological Wellbeing Practitioners [53].

Effectiveness studies of individual programs have demonstrated that, with appropriate training and supervision, a wide range of people can provide coaching. In one study, coaches were drawn from a mixed group of clinicians (general medical practitioners, psychologists, medical specialists, nurses, and other allied health practitioners) [45]. Other studies have included coaches who were: general medical practitioners with additional mental health training [54,55]; primary care nurses and social workers [7]; psychologists working in private practice [44] and in a non-government mental health organization [56]; community-based psychologists and social workers [46]; paraprofessional graduate mental health workers [57]; peer workers [58], and administrative staff [59].

There is great interest in incorporating the perspectives of people with lived experience of mental disorders into symptom-focused e-mental health programs. In addition to developing direct coaching roles, programs have included service user narratives in the automated content and/or hosted online peer forums within the program [59,60]. We are also aware of projects in which peer workers and advocates have developed their own coaching procedures for freely available Internet-delivered programs, in order to offer services to people living in areas that lack mental health treatment services.

##### What Knowledge is Required to Use the Coaching Model?

Providers already in clinical roles will have mental health knowledge and skills appropriate to their role and are able to provide mental health assessment, support, and referrals. Coaching that requires psychotherapeutic skills is likely to be delivered by providers who have therapy qualifications and already provide psychological services. In addition, they will need sufficient familiarity with e-mental health programs to match them with individual service users and skills relevant to providing coaching for the particular programs they use.

Coaches will also need to use their existing clinical skills, their understanding of particular programs, and service user preferences to decide whether to offer a coached intervention to an individual service user. Many protocols have excluded people who are psychotic, acutely suicidal, engaging in substance abuse, or have other conditions that may impair learning. Other factors to be considered include service users’ Internet access; comfort and proficiency in using text-based material and computer programs, and their capacity to work independently [61-63].

##### Current Status of the Coaching Model

###### Overview

Coaching protocols have been developed for specific programs and suites of related programs. They have largely been applied in relatively controlled settings such as research trials, centralized online clinics and university or hospital-based clinics (eg, [31,52,60,64]).

Compared to providers in these settings, community-based providers have more diverse qualifications, work in more diverse service settings and provide services to more diverse users. Rather than using a single program or programs from a single provider, they may wish to access a broad range of programs from various online providers for service users with diverse concerns and demographics.

Accordingly, examples of coaching protocols in the literature will often not, by themselves, be sufficient to guide providers in routine primary care settings. Rather, flexible protocols and clinical guides are required that can be tailored to the requirements of particular service users, the programs most relevant to those service users and the expertise of the service provider.

Community-based service providers who wish to participate in coaching activities can currently access protocols in two ways. First, some online services actively support community-based providers to coach users through the particular programs offered by that service. Resources available vary across programs but may include coaching guidance or manuals (eg, [47,52,65]). Training may be available for service providers to help them become familiar with the program, its coaching protocol, online communication techniques, and relevant ethical, legal, and technical issues [52,63].

Some e-mental health resources are explicitly designed to guide both the therapist and the service user to work on the material together during sessions. These do not necessarily reduce the amount of time required of service providers. Instead, they are designed to improve intervention quality and support a wider range of non-expert providers to deliver basic psychological interventions. For example, the Calm Tools for Living program guides the delivery of multi-diagnostic CBT treatment in primary care by health workers such as nurses and social workers [7]. Other examples include the StayStrong iPad app, which is designed for use with Australian Indigenous service users. It supports a wide range of workers to deliver a culturally appropriate structured mental health and substance abuse intervention [66].

Second, existing protocols (for example, those used in research) can be adapted to suit community-based providers and the service users they work with most frequently. The following draws on the research literature to illustrate the ways in which coaching can be implemented.

###### a) The Scope and Intensity of Coaching

Basic coaching activities include monitoring/ supervision, emotional and technical support, and encouragement to complete the program. Other coaching activities require psychotherapeutic skills, although the online program remains the primary focus. These include tailoring the program in response to individual service users’ progress (for example by controlling the release of new content) and helping them to complete critical tasks such as exposure, which they might otherwise avoid [67-69]. In the CALM program, coaches also demonstrate skills such as controlled breathing [7].

Further research is needed to identify which coaching activities are critical but some key considerations have been identified.

Qualitative interview data has underlined the importance of interpersonal relatedness for maintaining engagement with iCBT [70]. With regards to the coach/ service user relationship, the Supportive Accountability model is an overall model for optimizing human support in e-mental health interventions. It was proposed to aid research, but is a useful conceptual aid for practitioners entering this area [71].

Examples of coaching behaviors are available from analyses of emails sent by coaches to service users. In one study of an online program for Generalised Anxiety Disorder, the most frequently-coded therapist behaviors involved prompting and reinforcing task completion, and real-life application of program material. Reinforcement of task completion was significantly correlated with better outcomes. Poorer outcomes were associated with therapists being more flexible about program deadlines, but the direction of causality was not clear [72].

Similar themes were found in an analysis of therapist communications in a case study of iCBT for depression. Additional disorder-specific therapist communications were found, such as email content intended to increase hope and normalize the client’s experience [62].

###### b) The Amount of Time Required

Program protocols usually indicate the coaching time required but this may be minimal once the service user has begun working through the program (eg, [59,73]). Indeed, many coaching functions can be at least partially automated, for example reminders to complete tasks, feedback about symptom quizzes, and prompts to seek professional advice [56,59,68,74-76].

Providers should carefully monitor service users’ satisfaction with the time provided. Users may feel isolated and unmotivated if the coaching is too brief [70,77], although increasing amounts of therapist time may not necessarily improve outcomes [78]. Generally, however, more time will be required for coaching that involves therapeutic activity and coaching time in community settings will need to respond to the needs of individual service users [7,62,70].

###### c) Contact Arrangements

Service users can access online programs from locations of their own choice or from spaces provided by the coach (eg, [7,58]). Most symptom-focused programs require users to register an account. Some can be completed anonymously while others require users to provide personally identifying information.

In many protocols, contact between coaches and service users is asynchronous and conducted via emails, but may also occur through face-to-face meetings and telephone calls (eg, [44-46, 52, 54-56,64,66,79]). The use of text-based media such as email and chat requires additional training to ensure that the coach is competent in the clinical use of the media as well as secure online spaces in which to communicate with service users [26,46,80]. Coaching protocols usually provide email templates that coaches can personalize for individual service users (eg, [26,63,80]).

Most protocols specify deadlines for service users to complete tasks and schedules for contact between service users and providers, although service users may initiate additional contact [44-46]. Additional automated or human contact may be initiated at critical times, such as when users have not made contact within a specified time period, if coaches are concerned about users’ engagement with the program or their clinical status and when service users begin or complete key tasks [12,45-47,54,56].

Most programs provide service users with feedback as they work through the intervention, for example via symptom quizzes and other interactive exercises. Service users can print out this feedback and bring it to sessions or email it to their coach. Some programs provide information about user progress and clinical status directly to coaches [45,47,56,60,61].

### Integrative Models

#### Overview

In the previous three referral models, the e-mental health program is the main intervention and the content and structure of the intervention is determined by the e-mental health resource. Models 4 and 5 are integrative models, where the therapist determines the content and structure of the assessment and overall intervention.

e-mental health resources can be integrated into any part of the therapy process. During assessment, therapists may incorporate information from online assessments, which are available as stand-alone tools or embedded within information sites or symptom-focused programs [81,82]. These resources can also be used to help improve therapy readiness and engagement. Service users can be encouraged to access particular sites and to talk with the therapist about the sites they have visited. This may help normalize and destigmatize service users’ internal experiences, assist them to reflect upon and describe their internal experiences, help form optimistic and realistic expectations about therapy, and assist the transition into intervention. Anecdotal data indicates that the pre-therapy use of e-mental health resources may be particularly helpful with young people. Once a service user is engaged in treatment, providers may integrate e-mental health resources into their work to maximize the effectiveness of face-to-face sessions.

Two integrative models are described below. The first relates to integration of e-mental health resources with symptom-focused treatment, and the second refers to complex interventions.

#### 4) Integrated into Symptom-Focused Treatment

##### What is the Symptom-Focused Model?

In this model, the therapist provides an individualized assessment, formulation, and intervention that incorporates traditional therapeutic activities as well as e-mental health resources.

The relative contributions of electronic resources and traditional therapy will vary across therapists and service users. E-mental health resources can be used to assist with standard therapeutic tasks such as assessment, psycho-education, self-monitoring, cognitive restructuring and the development of skills such as relaxation and mindfulness. Online peer groups can provide opportunities for behavioral experiments and social skills practice exercises.

Symptom focused programs can also be incorporated into therapy by presenting material in both electronic and face-to-face formats. In one study, participants received face-to-face CBT therapy in the first half of the sessions and worked independently through an online program in the second half. Outcomes for anxiety, distress, and depression in the combined (face-to-face and online) intervention were as good or better than outcomes in the traditionally delivered (face-to-face only) intervention [83]. The combined intervention thus reduced therapist time and maintained or improved outcomes.

e-mental health and therapist-delivered material can also be delivered sequentially. For example, a service user could meet with a therapist for a series of face-to-face sessions and then complete a symptom-focused e-program before returning to the therapist for a follow-up assessment (cf [34]).

There is also potential to develop symptom-focused group therapy interventions using e-mental health programs. This could occur in a similar manner to that reported for a primary-care mood-disorders group based on a computerized (but not Internet-delivered) CBT program [84]. This group was hosted by a general medical practice that provided a meeting room and a computer for each participant. The facilitators set a module for each session and in the first part of the session each participant used their computer to work through the module. After a tea break, facilitators led a group session on the topic of the module that had been completed by the group members.

##### Which Providers Are Most Likely to Use the Symptom Focused Model?

This model is likely to be used by therapists already providing discrete symptom-focused psychological interventions such as manual-based CBT to relieve specific symptoms and disorders. In the Australian primary health care system for example, this model is particularly relevant to providers delivering Focused Psychological Strategies interventions under the Medicare Better Access program [85]. These providers include psychologists, social workers, occupational therapists, and general medical practitioners with relevant therapy training. It would also be relevant to mental health nurses and other mental health workers delivering therapy in other primary care services.

##### What Knowledge is Required to Use the Symptom-Focused Model?

In order to use the symptom-focused model, providers require existing qualifications and experience in providing face-to-face symptom-focused therapies. In addition, they would need to be sufficiently familiar with specific e-mental health resources to choose relevant elements and integrate them into bespoke interventions for individual service users.

##### Current Status of the Symptom-Focused Integration Model

Although there are anecdotal reports of this model’s use and some promising research data, more ecologically valid research is needed to evaluate whether it delivers improved effectiveness and efficiency in routine care.

#### 5) Integrated Into Comprehensive Therapy

##### What is the Comprehensive Therapy Model?

In this practice model, e-mental health resources are used in a comprehensive intervention designed and structured by the human therapist. The e-mental health resource is one element of a multi-modal approach that uses a range of interventions to treat a presenting problem or simultaneously addresses a number of presenting problems.

The therapist’s role will include both traditional therapeutic activities and activities related to the service users’ interaction with e-mental health resources. Therapists’ roles in relation to e-mental health will vary from case to case and may resemble activities in described Models 2, 3, or 4. Thus the therapist may encourage the service user to use an information site, online support group or symptom-focused program in a relatively independent manner and monitor the service users’ response to the resource. In other cases, the therapist will be more directly involved in supporting the service user’s interaction with the electronic resource. For example, they may provide some coaching for an online CBT program or integrate the program into a symptom-focused intervention.

This integrated comprehensive model can be used where multiple therapeutic approaches are required to tackle one presenting problem, particularly where first line interventions have been unsuccessful or where some components of therapy require an emphasis on interpersonal processes occurring in the therapeutic relationship. For example, the early stages of treating complex trauma may require a strong focus on building the therapeutic relationship and engagement. Concurrent use of e-mental health programs at this stage could help lay the foundation for challenging activities such as exposure work. For example, working through a symptom-focused program could help provide some initial symptom relief, improve skills for managing arousal and enhance stability and resilience.

This approach can also be helpful where there is more than one presenting problem, or where there are comorbidities and secondary disorders that require treatment. Given that the number of funded sessions is likely to be limited, augmenting human delivered therapy with e-mental health resources may reduce the risks associated with incomplete or inadequate treatment of complex issues.

##### Which Providers Are Most Likely to Use This Model?

This model will be most relevant to therapists who already provide comprehensive individualized therapies in their existing practice. In the Australian primary care system for example, this would include clinical psychologists providing psychological therapies under the Medicare Better Access program and other therapists whose work involves tackling complex problems. It would also be relevant to providers whose primary role is to treat very specific concerns and who may need an additional resource to tackle comorbid problems such as anxiety, depression, and substance abuse. For example, in relationship interventions where one or more of the participants is experiencing a high prevalence disorder such as depression, the therapist can focus on interpersonal processes during sessions and refer the symptomatic partner(s) to an online service that treats depression.

##### What Knowledge is Required to Use the Comprehensive Model?

Service providers who are engaging in comprehensive individualized assessment and therapy will already have extensive knowledge of psychological processes and diverse therapies as well as skills in developing multi-modal assessment and treatment plans. Those who provide therapy for specific issues such as relationship problems will have existing skills in screening for comorbid problems that require treatment. In addition, providers need to have familiarity with the e-mental health resources they intend to integrate into their therapies.

##### Current Status of the Comprehensive Therapy Model

At this stage, individual therapists are integrating e-mental health idiosyncratically into comprehensive therapies. Anecdotal evidence indicates that uptake of this model may be aided by its conceptual similarity to the use of face to face delivered CBT-based skills groups traditionally provided alongside individual specialist therapy in secondary care psychiatric services.

## Discussion

Community-based service providers are developing innovative ways of incorporating e-mental health resources into their practice. The models described here are intended to help researchers, policy-makers, and service providers conceptualize the ways in which e-mental health resources can be incorporated into existing systems of health delivery.

These practice models can also be integrated into innovative service delivery models that improve the delivery of mental health services in primary health care. For example, stepped care and stratified care models are distinguished from usual care partly by the systematic inclusion of less specialist and lower intensity psychological interventions [86-88]. The promotion, case management and coaching models can be used to deliver psycho-education, support, and symptom-focused interventions in low intensity interventions [89]. The integrative models described here can be incorporated into higher intensity interventions.

Similarly, collaborative care models for severe depression typically include case management or care coordination roles and systematically support service user self- management. Access to psychological interventions within collaborative care models is associated with better outcome [90]. Clearly, the incorporation of e-mental health resources could support this work, for example through the promotion of high quality online self-help material and case management referrals to iCBT.

The models described here are also compatible with emerging enhancements to service delivery systems. For example, virtual clinic environments are currently being developed that will offer a range of e-mental health resources within a defined online environment. They will provide a framework within which service providers and users can choose interventions of known quality and may allow the automation of some activities currently undertaken by humans, including aspects of promotion, case management, assessment, and coaching.

e-mental health resources support self-management and service user expertise and are thus also compatible with emerging recovery-focused, person-centered approaches. One example is the support facilitation approach currently being developed in Australia for people with complex, severe and persistent mental illness [91]. Support facilitation workers assist service users to engage with a range of services and the referral models (promotion, case management, and coaching) described in the current paper are highly relevant to these roles.

## Future Research

### Outcome Research

The current paper has demonstrated how e-mental health resources can be incorporated into routine care and some of the potential advantages of doing so. However, research into community providers’ use of e-mental health resources is at an early stage and it is not yet possible to determine the optimal ways for providers to interact with the resources. There is an urgent need for research to evaluate the effectiveness, safety, and efficiency of the clinical practice models described in this paper.

As an initial step, a systematic synthesis of existing literature is needed to identify gaps in the literature and inform clinical practice. The current paper is based on a narrative review in which the most visible body of e-mental health outcome research involved coaching, but a more systematic approach may identify additional bodies of evidence that relate to other practice models.

As outcome research develops in this area, it will be critical to use research methodologies that optimize ecological validity as well as scientific rigor. These should include practice-based methodologies that balance the need to retain intervention fidelity and the need for flexibility that allows interventions to be adapted for use in routine care settings [92]. It will also be important to actively involve practitioners in developing practice-based procedures to guide implementation and evaluation (eg, [56,93]).

### Research Into Providers’ Use of E-Mental Health Resources

Anecdotal reports and existing literature suggests that practitioners most commonly interact with e-mental health resources by recommending websites to service users. For example, nearly 90% of an Australian sample of general medical practitioners reported that they recommended websites to service users, and a mental health information site was included amongst the three most frequently recommended sites [94].

Other studies have investigated the uptake of iCBT amongst service providers and have reported low rates of use [61,95-97]. Providers seem to prefer the idea of adding iCBT to existing clinical practice rather than using it as a replacement. This preference has been reported in various provider groups including CBT therapists [97], youth workers [98], and rural health care practitioners [99].

Taken together, these studies suggest that community-based practitioners may be most likely to use online resources in ways that are most consistent with the promotion, case management, and integrative models described in the current paper. Unfortunately, as noted above, there is less research on these models than on the coaching model.

In order to assess the degree to which e-mental health resources are being integrated into health delivery systems, it will also be important to continue measuring uptake by provider groups. Some studies thus far have been limited by small sample sizes and a focus on measuring practitioner attitudes and intentions with less focus on quantifying actual behavior. Furthermore, the lack of clearly defined provider roles and behaviors in the use of e-mental health resources has meant that the meaning of “practitioner use” is at times unclear or variable across studies.

Future studies can be improved by the use of adequate samples and using the models proposed in the current paper to more clearly conceptualize and define the outcomes of interest.

### Research Into Factors That Influence Provider Behavior

Successful dissemination will require a good understanding of the factors that can hinder and facilitate provider engagement with online resources.

Existing surveys on practitioner attitudes to iCBT and have found that they are generally open to the concept of using iCBT, particularly for people with less severe or complex symptoms and where some clinical support is provided (eg, [98-102]). Factors related to technological proficiency and access do not seem to be major obstacles to uptake in general but are perceived to affect the viability of using e-mental health resources with specific clients, therapists, and settings [97,100,101].

Commonly cited barriers include providers’ lack of accurate knowledge about the resources, lack of training in their use, and concerns about their effectiveness, safety and clinical responsibility [97,98,100-105].

The Promotion Model requires relatively little change to providers’ usual practice, particularly if they already provide psycho-educational materials such as printed handouts. Practitioners who wish to use the other models described in this paper will need to gain new knowledge, engage more deeply with e-mental health resources, and make some conceptual changes to their practice.

Obstacles to acquiring relevant knowledge include having the time to review resources and lack of access to training [99]. Even where time and training is available, providers are unlikely to prioritize learning about e-mental health resources unless they perceive it to be beneficial. In the absence of direct financial incentives, potential benefits may include reduced expenses associated with preparing and printing psycho-educational material (promotion model), access to additional referral destinations (case management model), the possibility of achieving more within the same number of sessions (integrative models) and being able to meet the expectations of service users.

In Australia, evaluation activities associated with the eMHPrac project will contribute to future research. Data will be collected on the ways that providers use e-mental health resources and on the proportion of e-mental health service users who have accessed key websites as result of a recommendation from a provider. Annual surveys and evaluation of eMHPrac provider training interventions will provide data on factors that influence practitioner engagement with and use of online resources.

## Conclusions

e-mental health resources can be incorporated into the routine practice of diverse service providers and settings in primary health care. The current paper has described five clinical practice models to help researchers, policy-makers, and service providers conceptualize the ways in which e-mental health resources can be incorporated into existing and emerging systems of health delivery. Further research is urgently needed to identify the best procedures for implementing the various practice models, the best means of supporting practitioner uptake and to establish the effectiveness and safety of each practice model when applied in community-based routine care settings.
